# Biomarkers of resistance to radiation therapy: a prospective study in cervical carcinoma

**DOI:** 10.1186/s13014-017-0856-2

**Published:** 2017-07-17

**Authors:** P. Moreno-Acosta, A. Vallard, S. Carrillo, O. Gamboa, A. Romero-Rojas, M. Molano, J. Acosta, D. Mayorga, C. Rancoule, M.A. Garcia, M. Cotes Mestre, N. Magné

**Affiliations:** 1 Research Group in Radiobiology Clinical, Molecular and Cellular, National Cancer Institute, Bogotá, Colombia; 2 Research Group in Cancer Biology, National Cancer Institute, Bogotá, Colombia; 3 Department of Radiation Oncology, Institut de cancérologie de la Loire-Lucien Neuwirth, 108 bis, Avenue Albert Raimond, BP 60008, 42271 Saint-Priest en Jarez, France; 4 Unit of Analysis, National Cancer Institute, Bogotá, Colombia; 5 Group of Pathology Oncology, National Cancer Institute, Bogotá, Colombia; 6 Microbiology and Infection Diseases, The Royal Women’s Hospital, Melbourne, Australia; 7 Pathology Group, National University of Colombia, Bogotá, Colombia; 8 Group of Radiotherapy and Medical Physical, National Cancer Institute, Bogotá, Colombia

**Keywords:** Radioresistance, Biomarkers, IGF-1R, Radiation Therapy, Cervical Carcinoma

## Abstract

**Background:**

Clinical parameters and proteins have recently been suggested as possible causes of radiotherapy (RT) resistance in cervical carcinoma (CC). The objective of the present study was to validate prognostic biomarkers of radiation resistance.

**Methods:**

The present prospective study included patients undergoing RT with curative intent for histologically proven locally advanced squamous cell CC. Tissues and blood samples were systematically collected before RT initiation. Immuno-histochemistry was performed (IGF-IR α and β, GAPDH, HIF-1 alpha, Survivin, GLUT1, CAIX, hTERT and HKII). Response to radiation was assessed through tumour response 3 months after RT completion, through overall survival (OS) and through progression-free survival (PFS).

**Results:**

One hundred forty nine patients with a mean age of 46 years were included, with FIGO IIB (*n* = 53) and FIGO IIIB (*n* = 96) CCs. 61 patients were treated with exclusive RT + brachytherapy and 88 underwent chemo-radiotherapy + brachytherapy. Our findings suggest an association between hemoglobin level (Hb) (>11 g/dL) and 3 months complete response (*p* = 0.02). Hb level < 11 g/dL was associated with decreased PFS (*p* = 0.05) and OS (*p* = 0.08). Overexpression of IGF-1R β was correlated with a decreased OS (*p* = 0.007). Overexpression of GLUT1 was marginally correlated with reduced OS (*p* = 0.05). PFS and OS were significantly improved in patients undergoing chemoradiation versus exclusive radiotherapy (PFS: *p* = 0.04; OS: *p* = 0.01).

**Conclusions:**

IGF-1R β overexpression and Hb level (≤11 g/dl) were associated with poor prognosis, and thus appear to be possible interesting biomarkers of radiation resistance. Our results corroborate previous pre-clinical studies suggesting IGF-1R and hypoxia to be part of the biological pathways leading to radio-resistance.

## Background

In spite of screening campaigns, cervical carcinoma (CC) is still one of the most prevalent and lethal malignancy, especially in transition countries [1, 2]. When diagnosed at a locally advanced stage, concomitant chemotherapy associated with external beam radiation therapy (EBRT) and brachytherapy is considered to be the standard of care. However, 30–40% of patients with similar prognostic factors do not similarly respond to comparable standard treatments [3] probably because of a subpopulation of radioresistant tumor cells [4, 5]. However, the underlying biological phenomenon and the reasons of its variability from one patient to another are still unknown [6]. Different molecular factors involving tissue oxygenation, oncogene activation, loss of tumor suppressor genes and aberrant molecular signaling pathways (such as IGF-1R α and β, CAIX, GLUT-1, GAPDH, HIF-1 alpha, hTERT, Survivin and HKII proteins expressions) were recently identified in CC [3, 7–12]. Crosstalk between glucose metabolism and hypoxia were suggested, and could be the root of resistance to radiotherapy (RT). Warburg demonstrated in 1927 that most of cancer cells predominantly produced energy by a high rate of anaerobic glycolysis [13]. Recently, it was suggested that cancer cells widely expressed glucose-carrying membrane proteins (GLUT-1, GLUT-7), increasing neoplastic cells metabolism [7, 13–15]. Thus, GLUT-1 was reported to be overexpressed in 47% of CC cells [7, 16–18]. The overexpression of HKII, a glycolysis-related protein converting glucose to glucose 6-phosphate, was reported in 69.2% of CC cells [19]. Interestingly, HKII was shown to protect cells against oxidative stress, the main mechanism of radio-induced DNA damage and death. Moreover, the value of GLUT-1, HKII and GAPDH (another glycolysis-related factor) as endogenous marker of hypoxia was proven, with a correlation between increased expressions of proteins and tissue hypoxia [3, 7, 18, 20]. The overexpression of HIF-1 alpha, a protein induced by hypoxia that upregulates pro-survival and pro-proliferation signaling pathways, was reported to be a predictive marker of response and prognosis in locally advanced CCs treated with exclusive radiotherapy [21]. HIF-1 alpha also regulates CAIX, considered as an endogenous marker of hypoxia. CAIX overexpression was reported in 51% of CCs [18]. Insulin-like growth factor 1 (IGF-1), receptor (IGF1R), Survivin and Human telomerase reverse transcriptase (hTERT) were also related to these hypoxic and radio-resistant phenotypes in CC cells [3, 12, 22, 23]. Finally, higher levels of expression of GAPDH were observed in patients co-expressing IGF2 and IGF1R, with hemoglobin levels ≤11 g/dl. [3], highlighting the possible interaction between glucose metabolism and hypoxia induced factors.

Nevertheless, aforementioned analyses were performed in limited groups of heterogeneous patients, and results are still debated. It is of primary interest to identify prognostic biomarkers of response to radiation since targeting these pathways may directly lead to improve outcomes of RT in advanced-stage CC patients. The aim of the present study was to prospectively assess if the expression of proteins of interest (GLUT-1, HKII, GAPDH, HIF-1 alpha, CAIX, IGF1R α and β, hTERT, Survivin) and the pre-RT haemoglobin level could be used as reliable prognostic biomarkers of radioresistance, in a cohort of patients treated with radiation.

## Methods

The present prospective study was conducted at the National Cancer Institute (Bogota, Colombia), analyzing prognostic biomarkers in a cohort of patients treated with radiation. The institutional review board approved the study, which was conducted in compliance with the Helsinki declaration. Written informed consent was obtained from all patients before trial initiation.

## Patient population

Patients treated with RT or radio-chemotherapy for histologically proven squamous cell CC, with a FIGO stage IIB or IIIB, could be included. FIGO staging was based on clinical examination and imaging, with magnetic resonance imaging (MRI) and PET-CT. Patient and tumor characteristics (age, tumor size, haemoglobin level, FIGO staging, tumor differentiation, parametrial involvement, treatment type) were reported.

### Molecular techniques/protein expressions

Hemoglobin was systematically assessed before RT and radio-chemotherapy initiation. IGF-IR α and β, GAPDH, HIF-1 alpha, Survivin, GLUT1, CAIX, hTERT and HKII proteins expression was studied based on immunohistochemistry (IHC). Inmunohistochemical staining was performed before treatment on fresh tissue samples. The process of IHC was performed using the Dako kit in Vision + Dual Link system-HRP (Agilent Technologies, USA, Santa Clara, California). Tissue sections of 3 μm were deparaffinized, rehydrated and washed. Antigenic recovery was performed using a target antigen retrieval solution pH 9, 10X (Agilent Technologies, USA, Santa Clara, California). Tissues were placed in a 6% H_2_O_2_ solution for 7 min to block the endogenous peroxidase and then were washed with PBS. An incubation was performed for 45 min at room temperature with the following rabbit polyclonal antibodies: IGF-IRα (N-20: sc-712, Santa Cruz) at 1:40 dilution; IGF-IRβ (C-20: sc-713, Santa Cruz) at 1:50 dilution; CAIX (H-120: SC-25599, Santa Cruz) at 1:50 dilution, GLUT-1 (RB-9052-P, Thermo Scientific) at 1:100 dilution; HKII (ab37593, Abcam) at 1:50 dilution; GAPDH (NBP1–76693, Novus Biologicals) at 1:100 dilution, HIF-1 α (ESEE122, ab8366, abcam) at 1:400 dilution and Survivin (RB-9245-R7, Thermo Scientific) at 1: 50 dilution, hTERT (mAB telomerase Reverse Transcriptase ab5181 abcam) at dilution 1/20. IHC protein rating scales were based according to scales previously reported [7]. Assessment of each marker was carried out by two onco-pathologists. A strong expression was defined by a three crosses IHC staining and an expression ranging from 60 to 100%. A negative expression was defined by an absence of IHC staining and a < 10% expression.

### Treatment definition

A pelvic 3D conformational EBRT was planned to deliver 45 to 50.4 Gy in 25–28 fractions (1.8 Gy/day) in five weeks. A boost of EBRT was delivered to the parametrium in case of parametrial involvement, protecting the midline. After the initial EBRT, an intracavitary brachytherapy (high or low dose rate) was performed using cesium or iridium sources. The interval between EBRT beginning and brachytherapy completion was planned not to exceed 56 days [24]. Patients undergoing chemo-radiotherapy received cisplatin 40 mg/m2/week, during six weeks with an absolute maximum dose of 70 mg/week [24].

### Efficacy assessment

Response to treatment was assessed 3 months after RT completion, and every 3 months during the subsequent five years based on physical examination, computed tomography (CT) and MRI according to Response Evaluation Criteria In Solid Tumors (RECIST) version 1.0. Biopsies were performed in case of tumor persistence suspicion. The responder group was defined as the group of patients that presented complete response, and the non-responder group, as the patients that presented partial response, stable disease or tumor progression.

### Statistical analysis

Descriptive analyses were performed using measures of central tendency, location and dispersion for quantitative variables, and absolute and relative frequencies for categorical variables. For complete responses to radiation, bivariate analysis was performed using the Student t test, or nonparametric Mann–Whitney test if the assumption of normality was not met by the quantitative variables. For qualitative variables, the chi-square test or the Fisher’s exact test was used, if the number of assumption necessary to use the chi-square test was not met. Overall and progression-free survivals were defined as the time from treatment initiation to death from any cause and as the time from treatment initiation to relapse, respectively. Survival functions were built using the nonparametric Kaplan-Meier method. Survivals -according to the groups defined by the explanatory variables- were compared using the log rank test. The effect extent was calculated through the hazard ratio (HR) base on a univariate Cox regression model, verifying the proportionality assumption of the model. Variables studied in univariate analysis were used in a multivariate Cox analysis. Analyses were two-tailed. Type I error level was 0.05, except in case of multiple testing. In this situation, a correction was performed, based on the Dunn-Sidak’s method: α’ = 1-(1- α)^1/k^, k being the number of tests. Analysis of multiple correspondences was processed. Analyses were processed using the Stata 11 software (StataCorp LP, Tewas, USA) and the R 3.2.2 software (R Core Team. R Foundation for Statistical Computing, Vienna, Austria) with ADE4 and FactoClass packages.

## Results

### Patient and tumor characteristics

**Fig. 1:**
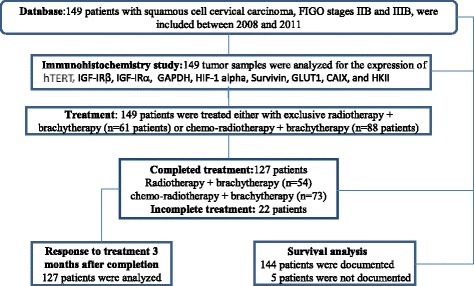
Flow-chart of the study. FIGO: Federation of Gynecology and Obstetrics; NCI, National Cancer Institute

A total of 149 patients with locally advanced squamous cell CC were included between 2008 and 2011, with a mean age of 46 years (range: 35–62). Patients had FIGO IIB (*n* = 53, 35.6%) and FIGO IIIB (*n* = 96, 64.4%) tumors. Most of patients (*n* = 117, 78.5%) presented a Karnofsky performance scale index >90%. Patient selection and characteristics are reported in Fig. 1 and Table 1.

**Table 1:** Patient and tumor characteristics

Characteristics	Mean (range)	Number of patients	%
Whole set of patients		149	100
Age, years	46.3 (26–75)		
Karnofsky index			
70–80		32	21.4
90–100		117	78.6
Histological type			
SCC		149	100
FIGO stage			
IIB		53	35.6
IIIB		96	64.4
Tumor size^a^			
<4 cm		8	5.4
≥4 cm		141	94.6
Tumor Differentiation			
Well		12	8.1
Moderately		101	67.7
Poorly		18	12.1
Undetermined		18	12.1
Haemoglobin, g/dL			
≤11 g/dL		102	68.5
>11 g/dL		47	31.5
Treatment type			
Exclusive EBRT + Brachytherapy		61	40.9
Chemo-Radiotherapy + Brachytherapy		88	59.1

*FIGO* International Federation of Gynecology and Obstetrics, *HPV* human papilloma virus, *SSC* squamous cell carcinoma, *EBRT* External beam radiation therapy

^a^Tumor size: Tumor size before treatment

### Treatment characteristics

Out of 149 patients, 61 patients experienced exclusive RT without concurrent chemotherapy and 88 underwent chemo-radiotherapy. A total of 22 patients (14.8%) did not receive the pre-planned treatment and therefore were not analyzed for efficacy 3 months after treatment completion (Fig. 1). EBRT doses ranged from 43.2 Gy to 55.8 Gy. Brachytherapy doses ranged from 25.0 Gy to 50.4 Gy to point A and from 6.6 to 35.0 Gy to point B. EBRT and brachytherapy were performed with a mean total duration of 6 weeks (range: 4–6).

### Hemoglobin and protein expression assessments

**Fig. 2:**
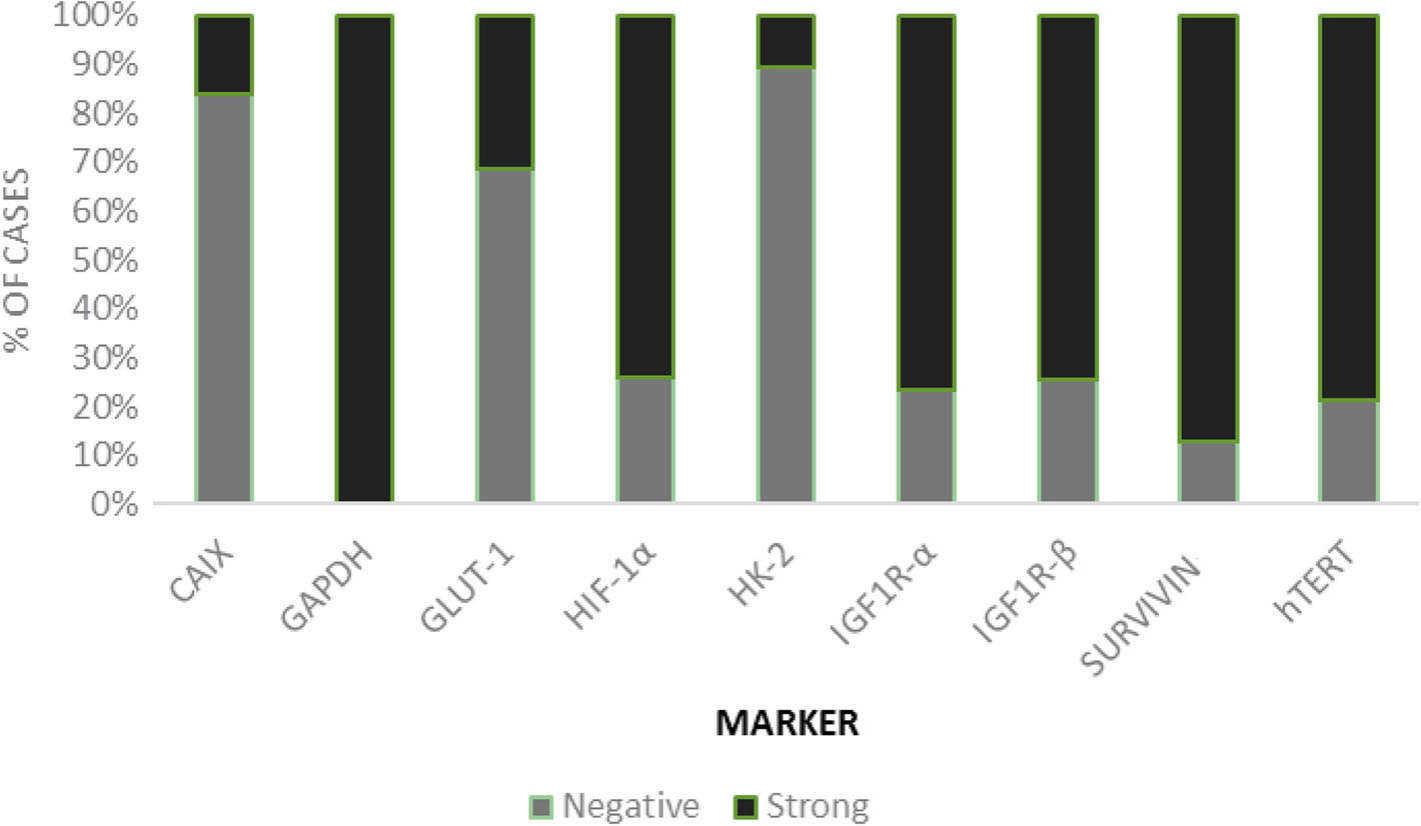
Assessment of protein expression in cervical carcinoma tissue

Mean pre-EBRT hemoglobin was 12 g/dL (standard deviation (SD) = 2.59; range = 3–16.9). Regarding immunohistochemistry analyses, the highest levels of protein strong expression were found in GAPDH (100%), Survivin (87%), hTERT (78.8%), IGF-IRα (76.5%), IGF-IRβ (74.5%), concomitant IGF-IRα and IGF-IRβ (73%), and HIF1α (74.1%). A negative expression was mainly reported with HKII (85%), CAIX (82%), and GLUT-1 (64%). Detailed results of protein expression within CC tissue are reported in Fig. 2.

### Data on efficacy: prognostic factors of early response to treatment

**Fig. 3:**
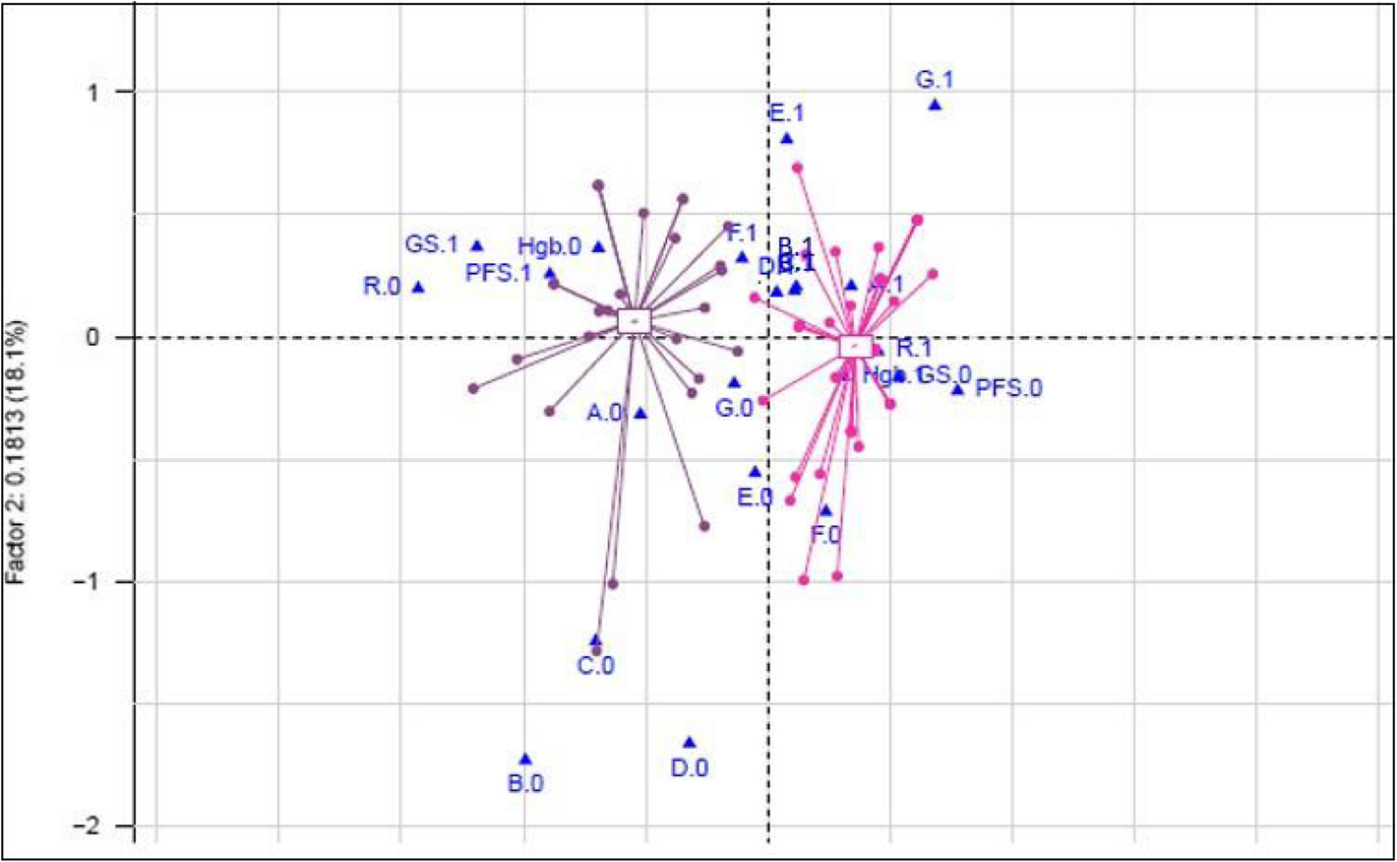
Multi-Correspondence Analysis (*n* = 71 patients included, for which all information was available). Legend: A: Type of treatment: Radiochemotherapy (1) and Radiotherapy (0); B: Survivin; C: IGF1R-β; D: IGF1R-α; E: GLUT1; F: HIF1-α; G: CAIX. For these: expression was Strong (1) or Negative (0); Hemoglobin (Hb): Hb > 11 g/dL (1); Hb ≤ 11d/dL (0); R0: non-responders; R1: Responders; GS.0: Alive GS1: Death PFS.0: No relapse PFS.1: Relapse

Correlation between biological and pathological characteristics, and 3-months-response to treatment was analyzed, showing a significant association of pre-EBRT haemoglobin > 11 g/dL and a complete 3-month-response (*p* = 0.02). The overexpression of proteins was not significantly correlated with complete response (type I error set to 0.0085 because of multiple testing). In a small number of patients, hTERT and HKII analyses could not be processed. Therefore, these proteins were not included in the analysis. Data on the comparison of responders and non-responders patients are displayed in Table 2, and Fig. 3 **.**

**Table 2:** Association between patient, tumor features and response to radiotherapy, 3 months after completion

Features	Responders	non-responders	Value - *p*
	(*n* = 92)	(*n* = 35)	
Median Age, years (SD)	47.5 (11.9)	49.9 (12.6)	0.17
Median Tumor size, cm (SD)	6.4 (2.0)	6.7 (2.1)	0.58
FIGO staging, number of patients (%)			0.26^*^
IIB	36 (78.3)	10 (21.7)	
IIIB	56 (69.1)	25 (30.9)	
Differentiation degree, number of patients (%)			
Well	7 (77.8)	2 (22.2)	0.62^*^
Moderetaly	65 (75.6)	21 (24.4)	
Poorly	11 (64.7)	6 (35.3)	
Treatment type, number of patients (%)			0.21^*^
Exclusive EBRT + Brachytherapy	36 (66.7)	18 (33.3)	
Chemo-Radiotherapy + Brachytherapy	56 (76.7)	17 (23.3)	
Protein Expression, number of patients (%)			
CAIX			
Negative	63 (70)	27 (30)	0.02^**₤^
Positive	19(95)	1 (5)	
HIF1 α			
Negative	27 (81.8)	6 (18.2)	0.14^*₤^
Positive	63 (68.4)	29 (31.6)	
GLUT1			
Negative	52 (72.2)	20 (27.8)	0.44^*₤^
Positive	30 (78.9)	8 (21.1)	
IGF1R α			
Negative	17 (65.3)	9 (34.7)	0.39^*₤^
Positive	73 (73.7)	26 (26.3)	
IGF1R β			
Negative	22 (75.8)	7 (24.2)	0.59^*₤^
Positive	68 (70.9)	28 (29.1)	
Survivin			
Negative	11 (78.6)	3 (21.4)	0.67^*₤^
Positive	74 (73.3)	27 (26.7)	
Impact of anemia, number of patients (%)			
Hb > 11 g/dl	71 (78)	20 (21.9)	0.02^*^
Hb ≤ 11 g/dl	21 (58.3)	15 (41.6)	

SD: standard deviation, FIGO: International Federation of Gynecology and Obstetrics, EBRT: external beam radiotherapy

^*^Chi2 test

^**^Exact fisher test

^₤^Type I error is set to 0.0085 in order to correct for multiple testing, based on the Dunn-Sidak’s method

### Data on efficacy: OS and PFS prognostic factors

The median follow-up was 2.1 years (range: 0.1–7.6). At the time of analysis, median OS and PFS were 2.1 years (range: 0.1–7.6) and 1.9 years (range: 0.1–7.6), respectively. At last follow-up, 31 patients (20.8%) had died: 20 exclusive RT patients and 11 chemo-radiotherapy patients. Chemo-radiotherapy was identified as a factor significantly improving OS and PFS. Indeed, median OS was 2.1 years (range: 0.2–7.6 years) for patients who underwent radical RT, and 2.2 years (range: 0.1–5.7 years) for patient who underwent radio-chemotherapy (HR = 0.36, CI95% (0.16–0.82), *p* = 0.01)). Median PFS was 1.8 years (range: 0.2–7.6 years) for patients who underwent exclusive RT, and 2.1 years (range: 0.1–5.9 years) for patient who underwent chemo-radiotherapy (HR = 0.57, CI95% (0.32–1.00), *p* = 0.04). IGF-1R β was correlated with survival, with a median OS of 3.2 years for patients without IGF-1R β over-expression vs. 2 years for the high-expression subgroup (HR = 5.4, CI95% (1.59–18.3), *p* = 0.007)). GLUT1 overexpression was marginally correlated with reduced OS (*p* = 0.05), with a median OS of 2.5 years for patients without over-expression vs. 1.9 years for the high-expression subgroup (HR = 2.22, CI95% (0.99–5.04)).

In a small number of patients, hTERT and HKII analyses could not be processed. Therefore, these proteins were not included in the analysis. Associations between clinic/pathological characteristics, biomarkers and OS/PFS are reported in Table 3 **.** Hemoglobin level (Hb ≤11 g/dl) was marginally correlated with reduced PFS (*p* = 0.05) and OS (*p* = 0.08). Interestingly, Hemoglobin level was not significantly correlated with Karnofsky index, based on Mann Whitney test (*p* = 0.65) and on Spearman test (*p* = 0.66). Furthermore, an analysis of multiple correspondences (Figure 3) was performed. Two distinct groups could be identified. A first group featured characteristics such as 3-months incomplete response, cancer relapse, death, Hb < 11 g/dL, treatment based on exclusive radiotherapy and HIF-1 alpha over-expression. The second group featured characteristics such as complete 3-months response, the absence of relapse, the absence of cancer-related death, the non-overexpression of HIF-1 alpha, Hb > 11 g/dl and a chemoradiation treatment. These results should be viewed with caution since results on HIF-1 alpha were not significant.

**Table 3:** Prognostic factors of progression free survival and overall survival

	Progression-free survival				Overall survival			
	Univariate Cox model		Multivariate Cox Model		Univariate Cox model		Multivariate Cox Model	
Variables	HR	95% CI	HR	95% CI	HR	95% CI	HR	95% CI
FIGO								
IIB	1.00				1.00			
IIIB	1.22	0.72–2.07	1.03	0.57–1.88	2.03	0.87–4.73	1.69	0.64–4.53
Differentiation degree								
Well	1.00				1.00			
Moderately	2.35	0.72–7.69	2.89	0.85–9.86	1.65	0.38–7.05	2.42	0.56–10.5
Poorly	1.78	0.45–7.03	1.83	0.46–7.50	0.92	0.15–0.56	0.95	0.15–5.70
Treatment type								
Exclusive EBRT + Brachytherapy	1.00				1.00			
Chemo-Radiotherapy + Brachytherapy	0.58	0.35–0.95	0.57	0.32–1.00	0.38	0.18–0.80	0.36	0.16–0.82
Tumor protein strong expression								
SURVIVIN	1.02	0.46–2.28	1.37	0.51–3.67	1.35	0.40–4.47	1.80	0.51–6.46
IGF1R-β	1.53	0.81–2.89	1.81	0.87–3.76	2.7	0.94–7.73	5.38	1.59–18.3
IGF1R-α	1.07	0.57–1.98	1.08	0.54–2.17	1.58	0.60–4.12	2.06	0.70–6.12
GLUT-1	0.99	0.54–1.83	1.04	0.55–1.98	1.66	0.77–3.55	2.22	0.99–5.04
HIF-1α	1.46	0.79–2.70	1.56	0.80–3.05	0.92	0.42–2.00	0.94	0.41–2.17
CAIX	0.52	0.20–1.32	0.50	0.20–0.30	0.53	0.16–1.77	0.58	0.17–2.02
Impact of anemia								
Hb	2.20	1.33–3.65	1.78	1.00–3.15	2.11	1.04–4.33	2.01	0.92–4.40

*HR* Hazard ratio, *CI* Confidence interval, *FIGO* International Federation of Gynecology and Obstetrics, *EBRT* External beam radiotherapy

## Discussion

The present prospective study underlines important, albeit well known, results: chemoradiation is superior to radiation and anemia is a poor prognostic marker. Expression of IGF-1R and GLUT1 were associated with poor overall survival in multivariate analysis, and therefore appear to be possible interesting biomarkers of radiation resistance. However, such results on protein expression need confirmation in a larger cohort of patients. A possible limitation to our study was that outcome could have been mediated by the poor performance status of anemic patients in contrast to the hypoxic effect on tumor biology. However in our set of patients, no significant correlation was identified between hemoglobin level and Karnofsky index, suggesting that the poor prognosis value of anemia could not only been seen through the prism of the performance status. Furthermore, previous experimental and clinical studies suggested a direct association between anemia and a poor tumor oxygenation [20], limiting the radio-induced oxygen effect and therefore decreasing the efficacy of radiotherapy. In squamous cell carcinoma and especially in CC, the prognostic impact of anemia is well-established [3, 7, 20]. Our findings suggest that besides molecular biomarkers, hemoglobin could a reliable, inexpensive and easily accessible biomarker of radiation-resistance. Although the frequency of expression of IGF1R alpha and Beta in this study was very similar, it was observed that only IGFIR Beta significantly impacted OS. IGF-1R was already described as a predictive biomarker of OS and of poor response to RT in CC [12]. The IGF-1R expression was related to a 28.6 times higher risk of RT failure in CC patients HPV16 (+), suggesting the IGF-1R expression to be a biomarker of radioresistance [3]. Interestingly, Kilic et al. suggested that HPV-16 could interact with IGF-1R in cervical tumors, resulting in an increased radioresistance [25]. Zacapala et al. reported that Asian-American variants of HPV16 induced the overexpression of IGF-1R [26]. Therefore, HPV-16 variants could also be biomarkers of radioresistance and anti-viral drugs might act as agents restoring radio-sensitivity [27]. Even if HPV was not assessed in the present study’s population, the probability of HPV infection was high in our set of patients, since most of Colombian CC are HPV-positive [28, 29].

More importantly, the most significant marker of treatment failure in the present study was the absence of concurrent chemotherapy, inducing a significant OS and PFS decrease. A survival benefit of 12% was previously reported with the combination of chemotherapy + radiotherapy in CC patients [20]. The present findings clearly confirm that patients with locally advanced CC should undergo concomitant chemoradiation, in accordance with international guidelines. However, the identification of radioresistance biomarker (and therefore the identification of patients absolutely requiring concurrent chemotherapy) is certainly a major challenge for developing countries, since chemotherapies cannot be systematically paid. The exclusive RT that was sometimes performed in the present study provides a unique and “pure” model of radioresistance in CC and could be the missing link between in vitro studies and state of the art chemoradiotherapy studies that probably feature too many parameters to identify radioresistance causes [27].

Finally, although the association between hypoxia and poor response to RT has been widely described and is known as a common cause of RT failure [30–32], no efficient solution could be found yet to offer neoadjuvant treatment for patients with the highly hypoxic cancers. Identifying biomarkers of radio-resistance is therefore of primary interest since the standard treatment may be modified according to tumors’ radio-resistance status, testing radiosensitizing treatments only in patients with radioresistant tumors [25]. However, targeted therapies development is a long and expensive process that often makes new anticancer drugs not affordable for transition countries. Original alternatives could be found, testing drugs already widely used for other non-cancer indications but clearly interfering with cancer promoting elements, with interesting results in particularly in glioblastoma [33]. To our knowledge, such a process has never been performed in CC. Curcumin (diferuloylmethane) is derived from the rhizome of the tropical plant *Curcuma longa*. It interferes with a large number of cell processes, regulating the expression of inflammatory cytokines (e.g., TNF, IL-1), growth factors (e.g., VEGF, EGF, FGF), growth factor receptors (e.g., EGFR, HER-2, AR), enzymes (e.g., hTERT, COX-2, LOX, MMP9, MAPK, mTOR, Akt), adhesion molecules (e.g., ELAM-1, ICAM-1, VCAM-1), apoptosis related proteins (e.g., Bcl-2, caspases, DR, Fas), and cell cycle proteins (e.g., cyclin D1) [33–35]. Curcumin has been recently described in pre-clinical studies as a natural inhibitor of IGF-1Rβ and GLUT1 [34, 36, 37] and could be safely associated with chemotherapy and radiotherapy [38, 39]. A prospective phase II study will be designed in a near future, in order to evaluate the effect of curcumin as an inhibitor of IGF-1Rβ and GLUT1 when given before radiotherapy.

## Conclusion

Chemo-radiotherapy and anemia were identified as factors significantly impacting survival. Expression of IGF-1R and GLUT1 could be associated with poor prognosis, and therefore appear to be possible interesting biomarkers of radiation resistance. These findings could contribute to test individualized neoadjuvant treatment for cervical cancer patients over-expressing IGR-1R.

## Abbreviations

CAIX: carbonic anhydrase IX
CC: cervical carcinoma
EBRT: external beam radiation therapy
GADPH: glyceraldehyde-3-phosphate deshydrogenase
GLUT: glucose transporter
HIF: hypoxia inducible factor
HK: hexokinase
HPV: human papilloma-virus
HR: hazard ratio
hTERT: human telomerase reverse transcriptase
IGF-1R: insulin growth factor receptor 1
IHC: immunohistochemestry
RT: radiotherapy
